# Raspberry-like PS/CdTe/Silica Microspheres for Fluorescent Superhydrophobic Materials

**DOI:** 10.1186/s11671-016-1325-9

**Published:** 2016-02-29

**Authors:** Jinghui Chang, Linlin Zang, Cheng Wang, Liguo Sun, Qing Chang

**Affiliations:** Key Laboratory of Chemical Engineering Process & Technology for High-efficiency Conversion, College of Heilongjiang Province, School of Chemical Engineering and Materials, Heilongjiang University, Harbin, 150080 China; School of Electrical Engineering, Heilongjiang University, Harbin, 150080 China

**Keywords:** CdTe, Polystyrene microspheres, Particulate films, Fluorescence, Superhydrophobicity

## Abstract

Superhydrophobic particulate films were fabricated via deposition of raspberry-like fluorescent PS/CdTe/silica microspheres on clean glass substrates and surface modification. Particularly, the fluorescent microspheres were prepared by a kind of modified strategy, namely introducing poly (acrylic acid)-functionalized polystyrene microspheres and thiol-stabilized CdTe quantum dots into a hydrolysis reaction of tetraethoxysilane simultaneously. And through adjusting the reaction parameters, the polystyrene spheres with two particle sizes and three colors of CdTe quantum dots aqueous solution were obtained. Consequently, raspberry-like microspheres consist of polystyrene cores and the composite shells of CdTe quantum dots and silica. These microspheres possess a fluorescent characteristic and form a hierarchical dual roughness which was conductive to superhydrophobicity, and the hydrophobic tests also showed the contact angles of water droplets on the surface of the raspberry-like microspheres which were over 160° at room temperature.

**Electronic supplementary material:**

The online version of this article (doi:10.1186/s11671-016-1325-9) contains supplementary material, which is available to authorized users.

## Background

Considerable attention has been paid to the wettability of solid surfaces due to its importance in the deep understanding of interface chemistry and the precise manufacture of surface microstructures as well as its great potential for fundamental and industrial applications [1–4]. Unfortunately, because of the expensiveness, instability, and fragility of the related materials and end-product films, most of these cases have difficulty in practical applications [5]. In recent years, polymer/silica raspberry-like particles synthesized in hydrolysis reactions for large-scale superhydrophobic materials have aroused much interest [6–9]. Meanwhile, some jobs have been reported on the synthesis of aqueous CdTe/silica composite particles in a hydrolysis reaction to maintain the fluorescent stability of aqueous CdTe quantum dots (QDs) [10, 11].

Herein, after studying the methods for preparation of polymer/silica raspberry-like particles [9] and the synthesis of aqueous CdTe/silica composite particles [12], we combined the two hydrolysis reactions into a simple process to prepare raspberry-like microspheres by introducing poly (acrylic acid) (PAA)-functionalized polystyrene (PS) microspheres and thioglycolic acid (TGA)-stabilized CdTe QDs into a hydrolysis reaction of tetraethoxysilane (TEOS). The monodispersed PAA-functionalized PS microspheres were used as cores, and tiny silica particles with CdTe QDs enfolded and then assembled on the surface of PS microspheres to construct raspberry-like microspheres during the hydrolysis process. Subsequently, the particulate films were constructed by depositing these raspberry-like fluorescent microspheres on clean glass substrates. After surface modification with fluoroalkyl-silane (FAS), superhydrophobic surfaces were obtained.

## Methods

**Fig. 1:**
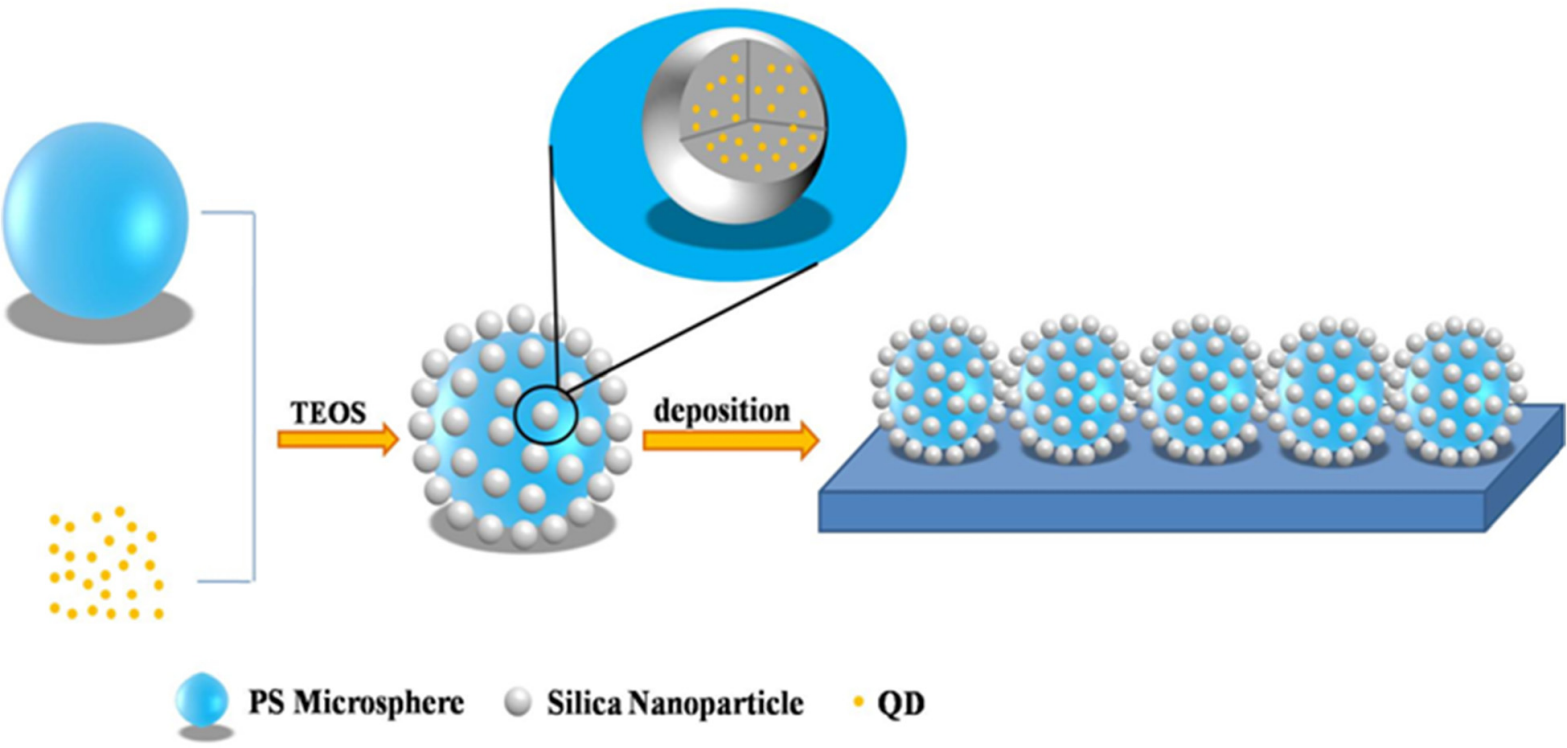
The formation process of the raspberry-like PS/CdTe/silica fluorescent microspheres and superhydrophobic film

The formation process of the raspberry-like PS/CdTe/silica fluorescent microspheres and superhydrophobic film were showed in Fig. 1. At first, PS microspheres with two different particle sizes were synthesized via the boiling soap-free method which was reported by Gu et al. [13], and three colors of CdTe QD aqueous solution were prepared according to the literature [14]. Subsequently, 161 mL absolute alcohol, 0.6 g PS microspheres, 3 mL ammonia, and 36 mL CdTe QD solution were added into a three-neck flask and stirred at 180 rpm under room temperature. The mixture solutions A (10 mL TEOS and 20 mL absolute ethanol) and B (0.9 mL ammonia, 18.3 mL absolute ethanol, and 10.8 mL deionized water) were poured in two syringes of a dual-channel pump and simultaneously dropped into the three-neck flask at a flow rate of 4 mL/h. After stirring for 3–5 h, a fluorescent microsphere suspension solution was obtained. The suspension solution was centrifugated with ultrasonic treatment repeatedly and then dispersed in deionized water. The as-prepared suspension solution was dripped on a clean glass substrate and dried in air. At last, after being sprayed and fluoridated by the FAS solution (1.0 g FAS, 50 g hexane, and 1.0 g distilled water with pH = 3 adjusted by acetic acid were mixed together and stirred at room temperature for 5 h before use), the fluorescent film with a superhydrophobic surface was prepared successfully.

The morphologies of CdTe QDs, PS microspheres, and PS/CdTe/silica fluorescent microspheres were characterized by TEM (FEITECNAI F20) and scanning electron microscope (SEM, S-4800), respectively. The crystallization of CdTe QDs was investigated by X-ray diffraction (XRD, D8 advance). Contact angle measurements for samples were performed using a contact angle meter (OCA20, Dataphysics) by placing a water droplet (~7 μL) on the surface of samples.

## Results and Discussion

**Fig. 2:**
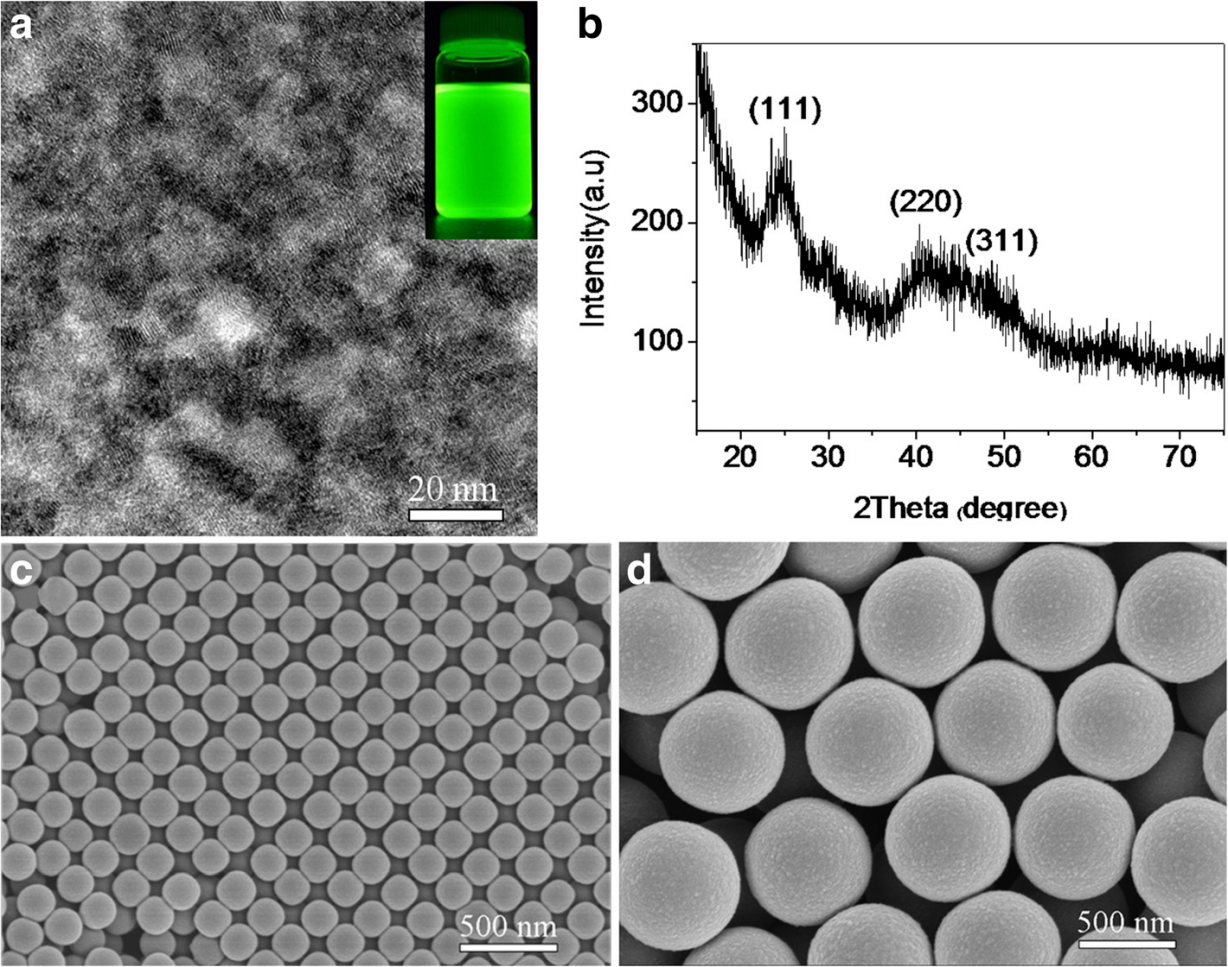
**a** TEM image of CdTe QDs with green fluorescence, and the *insert part* was its digital photo under UV rays. **b** XRD pattern of CdTe QDs with green fluorescence. **c** SEM image of PS microspheres (195 nm). **d** SEM image of PS microspheres (651 nm)

The TEM image in Fig. 2a showed that water-soluble CdTe QDs with green fluorescence had well-dispersed crystalline structures and irregular shapes and the fringe spacing of 0.22 nm corresponded to the interplanar spacing of the (220) cubic zinc blende [15]. In Fig. 2b, the diffraction peaks of CdTe QDs can be assigned to the diffraction planes of the cubic zinc blende structure of bulk CdTe crystal. Figure 2c, d were SEM images of PS microspheres with the diameters of 195 and 651 nm, respectively. Their associated standard deviations were 4.8 and 21.5 nm, respectively, which indicated the microspheres had well sphericality and uniform sizes and the polydispersity (PDI) data (nearly equal to 1.0) also indicated that the particle sizes were monodispersed according to the equations given by a former literature [6].

**Fig. 3:**
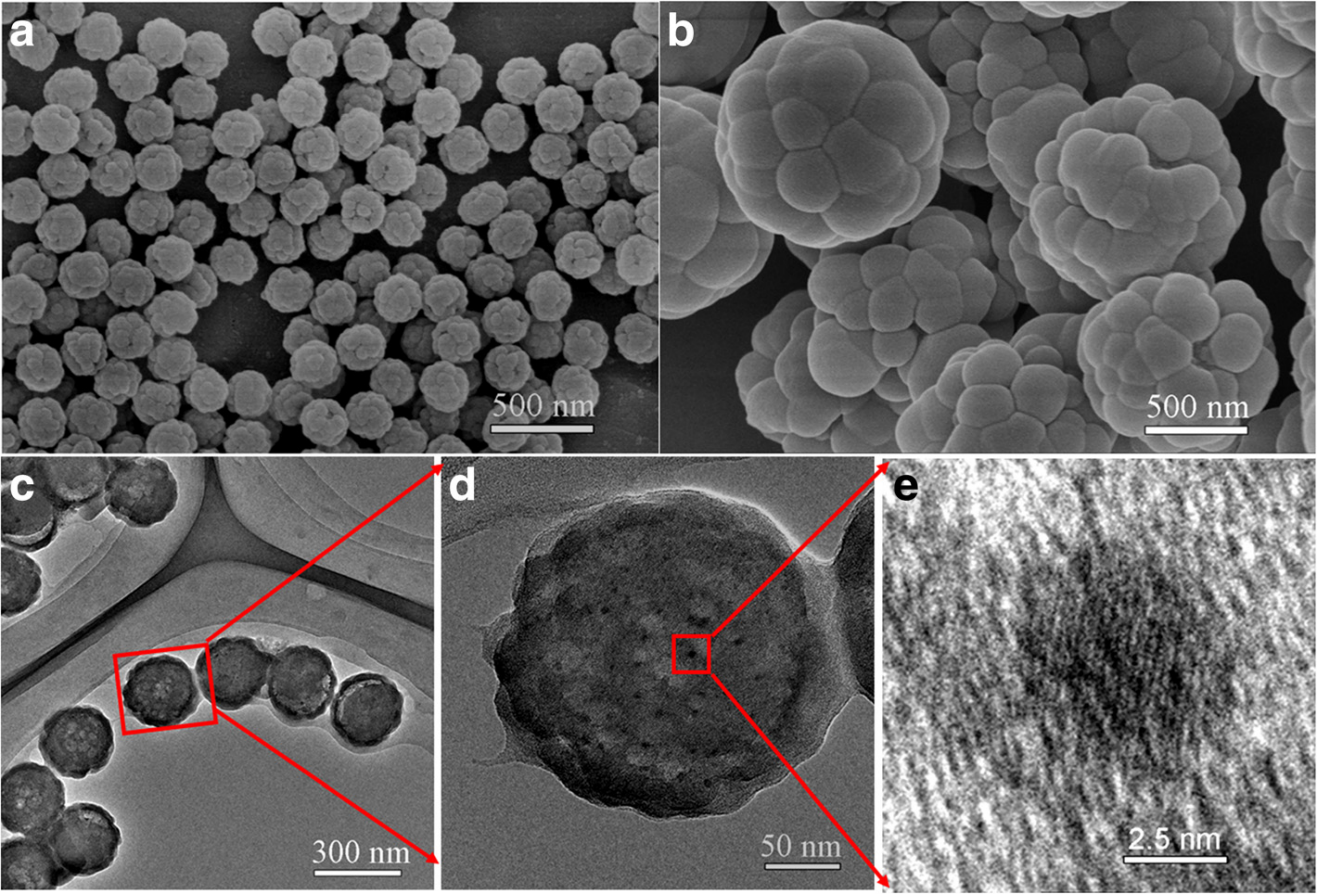
**a, b** SEM images of raspberry-like PS/CdTe/silica microspheres, and the diameters of PS cores were 195 and 651 nm, respectively. **c** TEM image and **d** magnified TEM image of PS/CdTe/silica microspheres with the core of 195 nm. **e** The TEM image of CdTe QDs in PS/CdTe/silica microsphere with the core of 195 nm

Figure 3a, b were SEM images of the raspberry-like PS/CdTe/silica microspheres which were formed via the use of the PS microspheres of 195 and 651 nm as the cores, respectively. Obviously, the microspheres exhibited raspberry-like morphology, and the tiny silica particles with different nanosizes were assembled on the surface of PS cores. The average diameters of the composite microspheres were about 239 and 1100 nm, and the size of the tiny silica particles increased greatly when the particle size of the PS microsphere changed from 195 to 651 nm. In addition, as shown in the SEM images, PS/CdTe/silica microspheres had well monodispersity corresponding with the polydispersity data (nearly equal to 1.0). Figure 3c–e were TEM images of the raspberry-like microspheres. It can be seen in Fig. 3e that the microspheres were about 241 nm in average size and CdTe QDs were about 5 nm, which was consistent with the conclusion in Fig. 2. When the average diameter of PS microspheres was 651 nm, TEM images of PS/CdTe/silicamicrospheres were shown in Additional file 1: Figure S1.

**Fig. 4:**
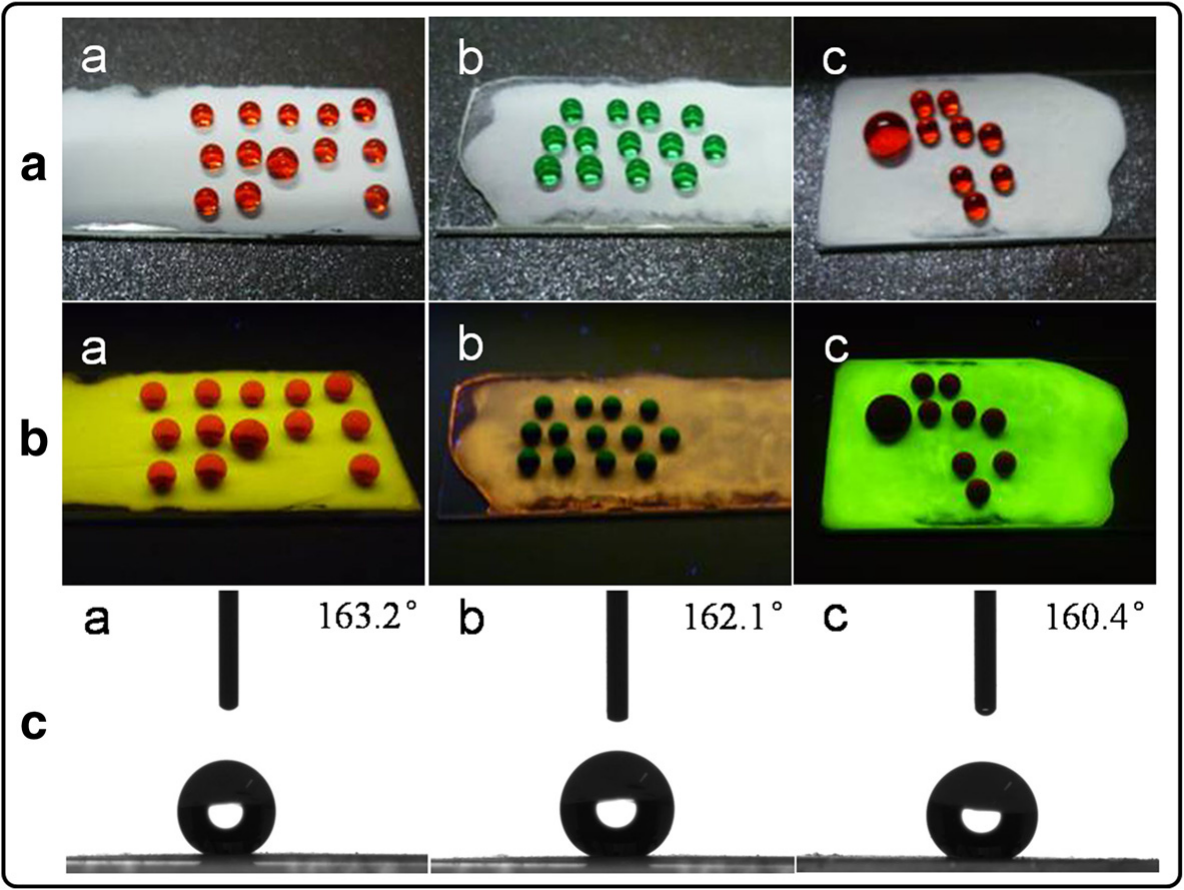
**a** Photographs of water droplets on particulate films under white light. **b** Photographs of water droplets on particulate films under UV rays, yellow (*a*), orange (*b*), and green (*c*). **c** The contact angle photos of water droplets on particulate films with PS cores (195 nm) and different fluorescent colors

Raspberry-like PS/CdTe/silica microspheres (the diameter of the PS core was 195 nm) were deposited onto the flat glass substrate to form the particulate film with hierarchical dual roughness. Followed by the introduction of FAS, the surfaces of the particulate film became superhydrophobic. These superhydrophobic films also displayed optical properties deriving from the CdTe QDs. As shown in Fig. 4a, b, the white particulate films exhibited yellow, orange, and green fluorescence under UV rays, respectively. After the hydrophobicity treatment, three kinds of fluorescent films possessed a well-hydrophobic property, and the contact angles of 7-μL water droplets measured on the surfaces of them were more than 160° at room temperature (Fig. 4c).

## Conclusions

In this study, the particulate films consisted of raspberry-like PS/CdTe/silica microspheres which were prepared via the use of functional PS spheres as the growing cores and the hydrolysis process. Due to the fluorescent characteristic of CdTe QDs and the modification with FAS, the films displayed three different colors under UV rays and their surface became hydrophobic. It is well known that the hierarchical structure was beneficial to hydrophobic property. Therefore, the contact angles of water droplets on particulate films could reach 163.2°. Furthermore, the materials for constructing this kind of dual-sized structured films have the advantages of low cost, stable fluorescence, and large-scale fabrication, which can make it a good candidate for the achievement of optical functional hybrid wettability surfaces.

## Additional File

Additional file 1: Figure S1. TEM images of PS/CdTe/silica microspheres with PS cores (651 nm).

## References

[1] Yao X, Song YL, Jiang L (2011). Applications of bio-inspired special wettable surfaces. Adv Mater.

[2] Feng XJ, Jiang L (2006). Design and creation of superwetting/antiwetting surfaces. Adv Mater.

[3] Dyett PB, Wu HA, Lamb NR (2014). Toward superhydrophobic and durable coatings: effect of needle vs crater surface architecture. ACS Appl Mater Interfaces.

[4] Li J, Jing ZJ, Yang YX, Yan L, Zha F, Lei ZQ (2013). A facile solution immersion process for the fabrication of superhydrophobic ZnO surfaces with tunable water adhesion. Mater Lett.

[5] Zhang X, Shi F, Niu J, Jiang YG, Wang ZQ (2008). Superhydrophobic surfaces: from structural control to functional application. J Mater Chem.

[6] Qian Z, Zhang ZC, Song LY, Liu HR (2009). A novel approach to raspberry-like particles for superhydrophobic materials. J Mater Chem.

[7] Hwang HS, Lee SB, Park I (2010). Fabrication of raspberry-like superhydrophobic hollow silica particles. Mater Lett.

[8] Wang RK, Liu HR, Wang FW (2013). Facile preparation of raspberry-like superhydrophobic PS particles via seeded dispersion polymerization. Langmuir.

[9] Lu Y, McLellan J, Xia YN (2004). Synthesis and crystallization of hybrid spherical colloids composed of polystyrene cores and silica shells. Langmuir.

[10] Zhu Y, Li Z, Chen M, Cooper MH, Lu GQ (M), Xu ZP (2012). Synthesis of robust sandwich-like SiO_2_@CdTe@SiO_2_ fluorescent nanoparticles for cellular imaging. Chem Mater.

[11] Shang HH, Gao XH (2010). Stable encapsulation of quantum dot barcodes with silica shells. Adv Funct Mater.

[12] Han K, Xiang Z, Zhao ZZ, Wang CL, Li MJ, Zhang H, Yan X, Zhang JH, Yang B (2006). Preparation of monodisperse CdTe nanocrystals-SiO2 microspheres without ligands exchange. Colloids and Surfaces A: Physicochem Eng Aspects.

[13] Gu ZZ, Chen HH, Zhang S, Sun LG, Xie ZY, Ge YY (2007). Rapid synthesis of monodisperse polymer spheres for self-assembled photonic crystals. Colloids and Surfaces A: Physicochem Eng Aspects.

[14] Gaponik N, Rogach LA (2010). Thiol-capped CdTe nanocrystals: progress and perspectives of the related research fields. Phys Chem Chem Phys.

[15] Li D, Wang YL, Xia YN (2003). Electrospinning of polymeric and ceramic nanofibers as uniaxially aligned arrays. Nano Lett.

