# Supplementary figures and images for: Raspberry-like PS/CdTe/Silica Microspheres for Fluorescent Superhydrophobic Materials
Source: Nanoscale Res Lett. 2016 Feb 29;11:114. doi: 10.1186/s11671-016-1325-9 (PMC4771649; doi:10.1186/s11671-016-1325-9)

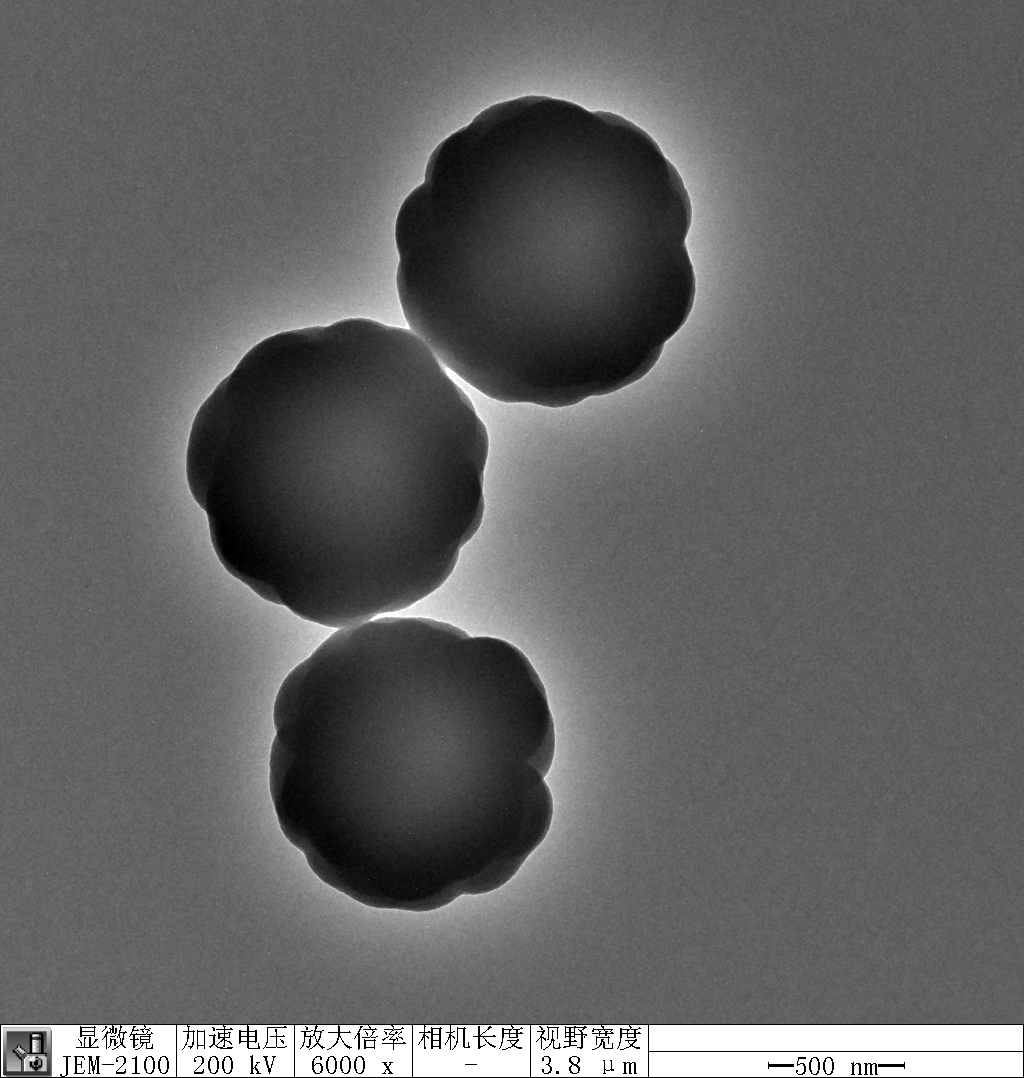

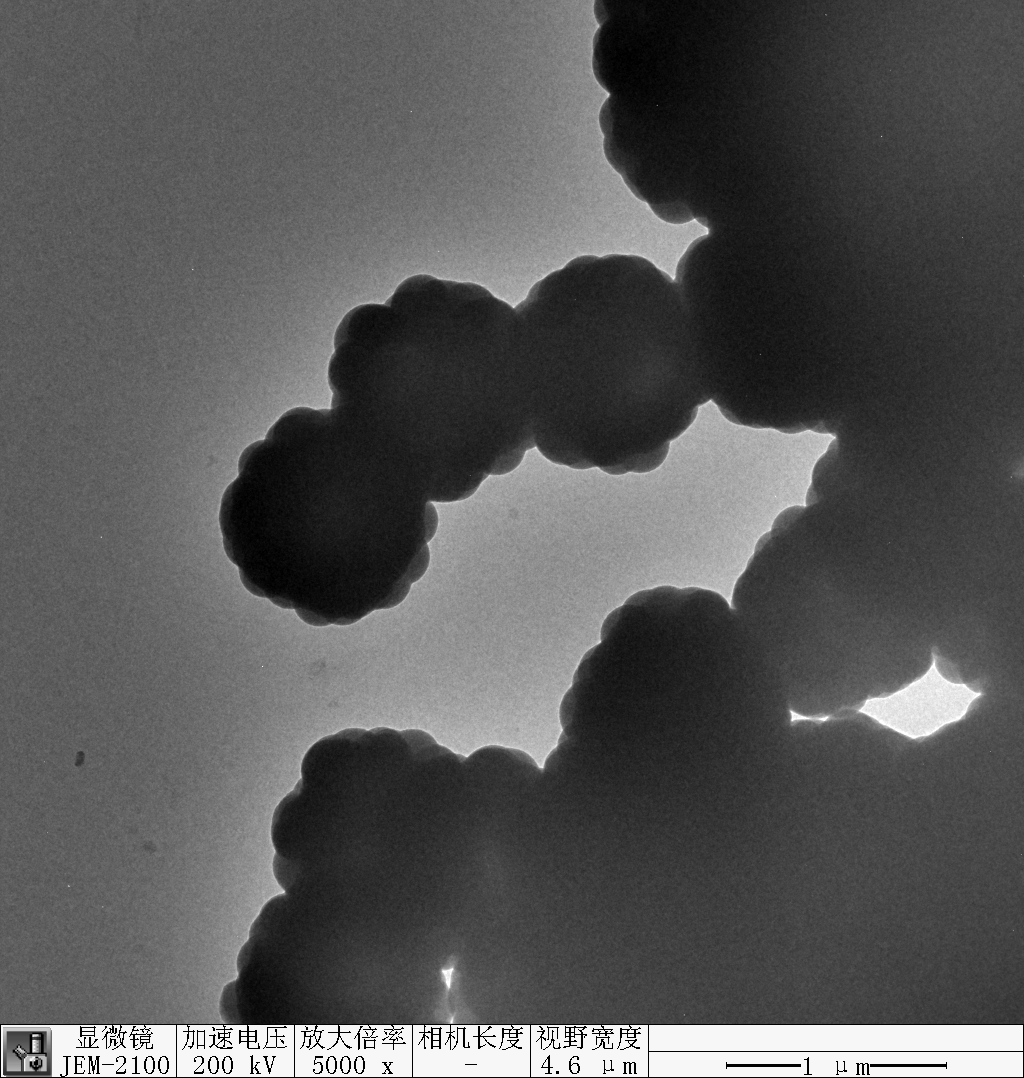


Additional file 1: Figure S1. TEM images of PS/CdTe/silica microspheres with PS cores (651 nm).

Supplement: Additional file 1: Figure S1. — TEM images of PS/CdTe/silica microspheres with PS cores (651 nm). [file 11671_2016_1325_MOESM1_ESM.doc]
